# Modification of Collagen by 3-Deoxyglucosone Alters Wound Healing through Differential Regulation of p38 MAP Kinase

**DOI:** 10.1371/journal.pone.0018676

**Published:** 2011-05-06

**Authors:** Danielle T. Loughlin, Carol M. Artlett

**Affiliations:** Department of Microbiology and Immunology, Drexel University College of Medicine, Philadelphia, Pennsylvania, United States of America; University of Hong Kong, Hong Kong

## Abstract

**Background:**

Wound healing is a highly dynamic process that requires signaling from the extracellular matrix to the fibroblasts for migration and proliferation, and closure of the wound. This rate of wound closure is impaired in diabetes, which may be due to the increased levels of the precursor for advanced glycation end products, 3-deoxyglucosone (3DG). Previous studies suggest a differential role for p38 mitogen-activated kinase (MAPK) during wound healing; whereby, p38 MAPK acts as a growth kinase during normal wound healing, but acts as a stress kinase during diabetic wound repair. Therefore, we investigated the signaling cross-talk by which p38 MAPK mediates wound healing in fibroblasts cultured on native collagen and 3DG-collagen.

**Methodology/Principal Findings:**

Using human dermal fibroblasts cultured on 3DG-collagen as a model of diabetic wounds, we demonstrated that p38 MAPK can promote either cell growth or cell death, and this was dependent on the activation of AKT and ERK1/2. Wound closure on native collagen was dependent on p38 MAPK phosphorylation of AKT and ERK1/2. Furthermore, proliferation and collagen production in fibroblasts cultured on native collagen was dependent on p38 MAPK regulation of AKT and ERK1/2. In contrast, 3DG-collagen decreased fibroblast migration, proliferation, and collagen expression through ERK1/2 and AKT downregulation via p38 MAPK.

**Conclusions/Significance:**

Taken together, the present study shows that p38 MAPK is a key signaling molecule that plays a significantly opposite role during times of cellular growth and cellular stress, which may account for the differing rates of wound closure seen in diabetic populations.

## Introduction

The wound healing process is a complex series of events that is characterized by several phases including inflammation, proliferation, and remodeling [1], [2]. One of the most important events in wound repair is the migration and proliferation of dermal fibroblasts resulting in wound closure. Infiltration of fibroblasts allows for the remodeling of the extracellular matrix (ECM) and retraction of the wound edges [1], [2]. Chronic cutaneous wounds which fail to heal within an expected time period are characterized by impaired fibroblast proliferation and migration within the wound area [1], [3], [4]. Impaired wound healing is a well-documented phenomenon in diabetes. Studies have shown that the hyperglycemic state in diabetes is accompanied by impaired wound repair as seen with decreased cellular migration and proliferation and decreased collagen production and matrix formation [1], [3], [4], [5], [6], [7], [8]. One possible reason for the defective wound healing capacity observed in diabetes could be the presence of the advanced glycation end product precursor 3-deoxyglucosone (3DG). We previously demonstrated that 3DG-modified collagen reduced fibroblast migration [9], proliferation, and ECM production [10] while increasing apoptosis through the activation of p38 mitogen activated protein kinase (MAPK) [11].

Many cytokines and growth factors transduce their signals via activation of various kinase pathways. Among these are the growth activated protein kinases including extracellular signal-regulated protein kinase (ERK), the protein kinase B (PKB/AKT), and the stress-activated protein kinase p38 MAPK. Both ERK and AKT play an important role in signaling during cell proliferation, while p38 MAPK has been classically associated with apoptosis and cellular stress [12], [13], [14], [15], [16], [17], [18]. Recent studies have begun to show an important role for p38 MAPK in cellular migration and proliferation [14], [19], [20], [21], [22], [23], [24]. In dermal fibroblasts, phosphorylation of p38 MAPK was found along the wound edge and correlated with cell migration into the wound [20]. Chemical inhibition of p38 MAPK resulted in decreased migration and increased apoptosis of wounded dermal fibroblasts suggesting p38 MAPK plays a positive role in regulating wound healing [20], [25]. Furthermore, pharmacological inhibition of p38 MAPK with SB202190 resulted in cell death under normal growing conditions and this death was attributed to the specific inhibition of the p38*β* isoform of p38 MAPK, while the p38α isoform was found to contribute to this cell death [26]. Despite the recent studies suggesting a growth stimulating role for p38 MAPK, a majority of the studies performed on p38 MAPK have attributed this kinase to cell stress and reduced proliferation. In support of this, we have shown that inhibition of p38 MAPK resulted in caspase-3 activation in dermal fibroblast cultured on native collagen, while it inhibited 3DG-collagen-induced caspase-3 activity [11]. This dichotomy suggests that p38 MAPK can be activated to signal as either a growth kinase or a stress kinase which is dependent upon extracellular stimuli.

The benefits of p38 MAPK signaling in wound healing is controversial. During normal wound healing, activation of p38 MAPK results in complete wound closure while in chronic diabetic wounds, p38 MAPK signaling results in aberrant wound repair [1], [8], [15], [20], [22], [24], [25], [27], [28]. One reason for this dichotomy could be the differential regulation of p38 MAPK during both normal and diabetic wound repair. Currently there is no known mechanism by which 3DG-collagen reduces proliferation and migration of dermal fibroblasts; therefore, it is important to understand the role of p38 MAPK during wound repair on 3DG-collagen. In the present study we examined the role of p38 MAPK signaling in dermal fibroblasts cultured on both native collagen and 3DG-collagen to address the role of this kinase in the regulation of wound healing.

## Results

### p38 MAPK differentially regulates the phosphorylation of ERK1/2 and AKT

**Figure 1:**
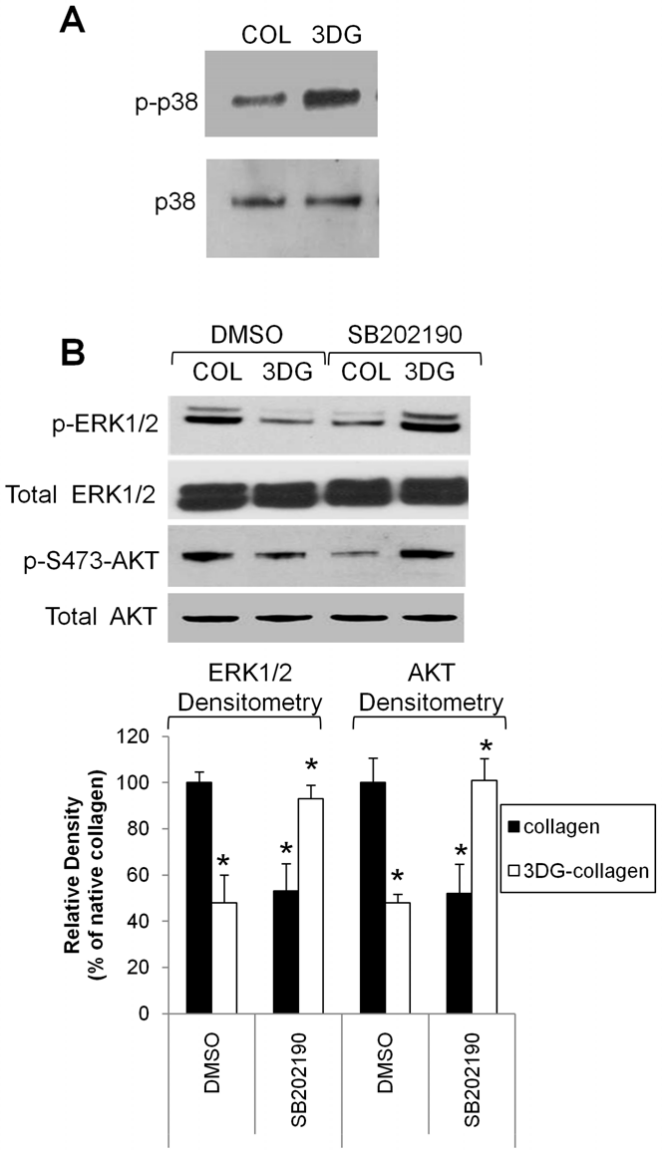
p38 MAPK differentially regulates ERK1/2 and AKT phosphorylation. 70% confluent fibroblasts were pretreated with SB202190 or vehicle DMSO for 1 h and cultured on native collagen or 3DG-collagen for 24 h. Expression of phospho-p38 MAPK (**A**), and phopsho-ERK1/2 and phospho-AKT, (**B**) were analyzed by Western blot using whole cell lysates. Total p38 MAPK, total ERK1/2, and total AKT served as loading controls. The bars correspond to the densitometric values of the intensity of both phospho-ERK1/2 bands and the phospho-AKT band compared to that of total ERK1/2 (both bands) and AKT band within each sample, respectively. The density of each sample was then made relative to the density observed in cells cultured on native collagen. All comparisons were made against their respective controls corresponding to native collagen treated with DMSO or 3DG-collagen treated with DMSO. Data are mean ± SD (n = 3), *P<0.01.

Previous studies within our laboratory have shown that 3DG-collagen can induce a sustained increase in the phosphorylation of p38 MAPK at 24 h [11]. Sustained p38 MAPK activity is responsible for much of the stress-induced apoptosis observed in cells, while low and transient phosphorylation of p38 MAPK is associated with cell proliferation and migration. Studies have shown that p38 MAPK can cross-talk with other pro-survival kinases, including ERK1/2 and AKT [14], [19], [29], [30]. During cell proliferation and migration, p38 MAPK, ERK1/2, and AKT are simultaneously upregulated [14], [19], [21]. However, during cellular stress, p38 MAPK activation correlates with reduced phosphorylation of ERK1/2 and AKT resulting in decreased proliferation and migration of the cell and increased apoptosis [16], [29], [31], [32]. Therefore, we investigated if 3DG-collagen-induced p38 MAPK signaling modulates the phosphorylation of ERK1/2 and AKT. Fibroblasts were pretreated with SB202190 (p38 MAPK inhibitor) or the vehicle DMSO for 1 h and then cultured on native collagen or 3DG-collagen for 24 h. Phosphorylation of p38 MAPK, ERK1/2, and AKT was detected by Western blotting. Confirming our previously published data [11], phosphorylation of p38 MAPK was increased by 75%±4.1% when fibroblasts were grown on 3DG-collagen compared to those cultured on native collagen (Figure 1A). Inhibition of p38 MAPK with SB202190 in fibroblasts cultured on 3DG-collagen increased the expression of phospho-ERK1/2 from 48%±12% to 93%±5.8% (Figure 1B, p<0.01). Likewise we observed that phospho-AKT increased from 48%±3.7% in cells cultured on 3DG-collagen to 101%±9.5% in fibroblasts cultured on 3DG-collagen with the inhibitor SB202190 (Figure 1B, p<0.01). However, in fibroblasts cultured on native collagen, we observed that inhibition of p38 MAPK downregulated phospho-ERK1/2 to 53%±12% and phospho-AKT to 52%±12.7% (Figure 1B, p<0.01) suggesting 3DG-collagen changes the cross-talk between p38 MAPK, ERK1/2, and AKT. These results also suggest that p38 MAPK in the presence of native collagen can act as a growth kinase promoting the phosphorylation of both ERK1/2 and AKT. However, the signaling in fibroblasts cultured on 3DG-collagen altered p38 MAPK to act as a stress kinase resulting in the depression of ERK1/2 and AKT phosphorylation.

### p38 MAPK inversely regulates the migration of fibroblasts cultured on 3DG-collagen or native collagen

**Figure 2:**
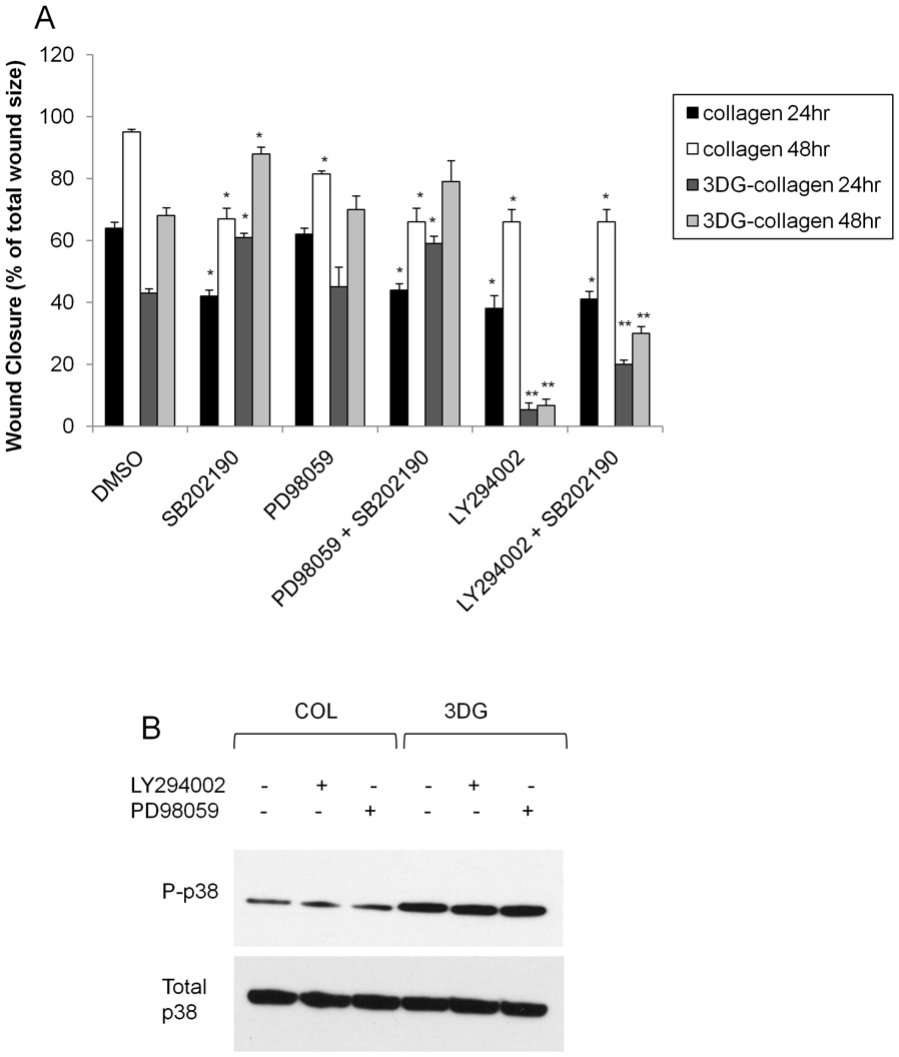
Wound closure rates in fibroblasts pretreated kinase inhibitors. **A.** Confluent fibroblasts were pretreated with the inhibitors; p38 MAPK inhibitor SB202190, AKT inhibitor LY294002, or the ERK1/2 inhibitor PD98059 for 1 h and cultured on native collagen or 3DG-collagen. The fibroblasts were then scratched manually with a pipette tip to introduce a wound as previously described. Cell migration into the wound was monitored at 0 h, 24 h, and 48 h by bright field visualization with an epi-fluorescence microscope. For each sample the distance across the wound margin was measured at 10 different points from wound edge to wound edge using Spot software. The measured distance was then converted into a percentage of wound closure when compared to the initial scratch at 0 h as shown in Material and Methods. All statistical comparisons were performed within each time point. Inhibitor comparisons were compared to their respective controls (native collagen or 3DG-collagen). **B.** To determine that LY294002 or PD98059 did not affect p38 phosphorylation, we pretreated fibroblasts with the AKT or ERK inhibitors for 1 h and cultured the cells on native collagen (COL) or 3DG-collagen (3DG) for 24 h. Whole cell lysates were extracted and phosphorylated p38 MAPK was detected by Western blotting. Phosphorylation of p38 MAPK was normalized to total p38 MAPK. The blot shown is representative of two individual experiments, each producing similar results. All statistical comparisons were performed within each time point. Inhibitor comparisons were compared to their respective controls (native collagen or 3DG-collagen). Data are mean ± SD (n = 3), **P<0.0001, *P<0.001.

We previously demonstrated that 3DG-collagen downregulated the migration of dermal fibroblasts in an *in vitro* wound site [9]. Since we observed that the inhibition of p38 MAPK in fibroblasts cultured on 3DG-collagen restored the level of phospho-ERK1/2 and phospho-AKT to levels observed in fibroblasts cultured on native collagen, and the phosphorylation of these proteins are known to promote the growth and migration of fibroblasts; we sought to evaluate the significance of p38 MAPK on fibroblast migration. Utilizing an *in vitro* scratch assay, confluent fibroblasts were pretreated with DMSO, SB202190 (p38 MAPK inhibitor), PD98059 (ERK1/2 inhibitor), or LY294002 (AKT inhibitor) for 1 h and a scratch was made along the monolayer of cells. The cells were cultured with the inhibitors for an additional 24 h or 48 h. Fibroblasts cultured on native collagen in the absence of any of the inhibitors had closed the wound by 95%±1.0% by 48 h, while fibroblasts cultured on 3DG-collagen had closed the wound by 68%±2.6% (Figure 2, p<0.001). In the presence of the p38 MAPK inhibitor SB202190, fibroblasts cultured on native collagen were unable to efficiently migrate into the wound, resulting in only 67%±3.5% closure by 48 h, the same as that observed in fibroblasts cultured on 3DG-collagen (Figure 2, p<0.001). However, inhibition of p38 MAPK in fibroblasts cultured on 3DG-collagen restored the migration of fibroblasts, closing the wound by 88%±2.2% in 48 h (Figure 2, p<0.001). These results suggest that p38 MAPK may be acting as a stress kinase in the presence of 3DG-collagen resulting in decreased migration, while p38 MAPK may act as a growth response kinase in fibroblasts cultured on native collagen allowing for fibroblast migration and closure of the wound.

To further investigate how p38 MAPK may be altering cellular migration, we investigated the growth kinases ERK1/2 and AKT, which are known to regulate wound closure. Fibroblasts pretreated with the ERK1/2 inhibitor PD98059 and cultured on either native collagen or 3DG-collagen did not alter the rate of wound closure compared to that seen in their respective control groups (Figure 2). Moreover, inhibition of both ERK1/2 and p38 MAPK resulted in wound closure rates similar to that seen in fibroblasts pretreated with only the p38 MAPK inhibitor, therefore wound closure is not significantly dependent on ERK1/2 activation. Pretreatment of fibroblasts cultured on native collagen with the AKT inhibitor LY294002, closed the wound by only 66%±4.0% in 48 h (p<0.001), and the addition of LY294002 to fibroblasts cultured on 3DG-collagen resulted in a significant further reduction in cell migration, as only 6.7%±2.1% of the wound was closed in 48 h (Figure 2, p<0.0001). The addition of the p38 MAPK inhibitor SB202190 did not further alter the wound closure rates of fibroblasts cultured on native collagen (66%±4.0%) suggesting that p38 MAPK dependent activation of AKT controls migration along native collagen. In contrast, p38 MAPK and AKT inhibition showed a partial upregulation in wound closure compared to AKT inhibition alone in fibroblasts cultured on 3DG-collagen (30%±2.2%) suggesting that p38 MAPK regulation of wound closure is primarily dependent upon the activation of AKT, however in the absence of both p38 MAPK and AKT, activation of ERK1/2 or other survival kinases may help promote migration (Figure 2). Furthermore we did not see any change in the phosphorylation of p38 MAPK after the use of the ERK and AKT inhibitors suggesting these inhibitors only affected the their own kinase activity and not that of p38 MAPK (data not shown). These results suggest that regulation of wound healing is dependent on p38 MAPK.

**Figure 3:**
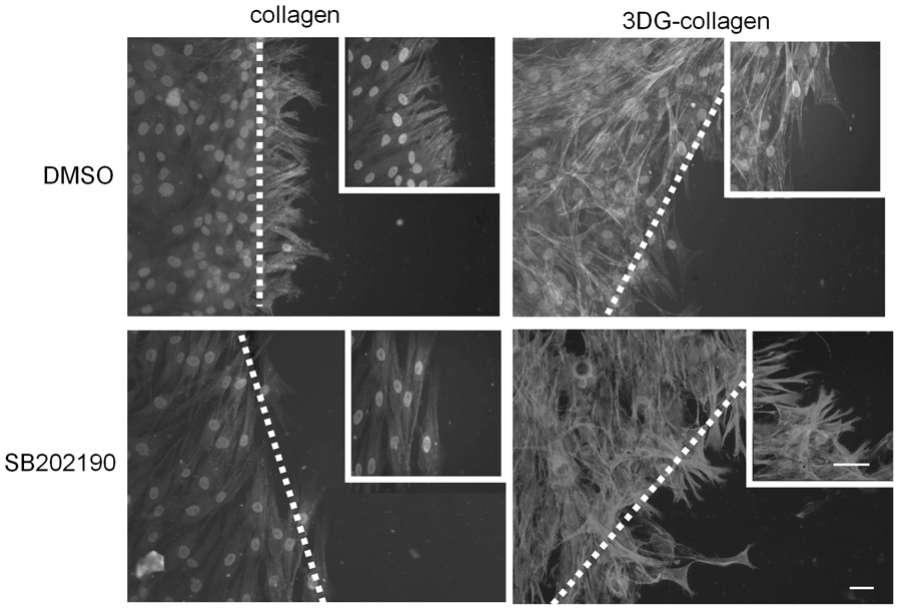
Differential regulation of filopodia extension after fibroblast pretreatment with the p38 MAPK inhibitor SB202190. Fibroblasts were pretreated for 1 h with the p38 MAPK inhibitor SB202190, or the vehicle DMSO and then cultured on native collagen or 3DG-collagen until confluent. The monolayer of cells was then manually scratched with a pipette tip to introduce the wound. At 4 h post-scratch the fibroblasts were fixed, permeabilized with Triton X-100, and stained with the F-actin dye rhodamine phalloidin. Extension was denoted as filopodia protrusion from initial wound site. The dotted line denotes initial wound site taken at 20 X magnification. Inset picture taken at 40 X magnification. Scale bar represents 10 µm.

One of the main migratory features of dermal fibroblasts is the extension of their filopodia along the collagen matrix [33], [34], [35]. After mechanical wounding, fibroblasts begin to extend their filopodia into the wound site by 4 h [9], [36]. We previously demonstrated that 3DG-collagen caused reduced filopodia extension [9]. Therefore, we investigated the effect of p38 MAPK on filopodia extension of fibroblasts cultured on native collagen and 3DG-collagen after mechanical wounding. Fibroblasts were pretreated with the p38 MAPK inhibitor SB202190 and the cultured on native collagen or 3DG-collagen until confluent. After confluency, a scratch was made and the actin filaments were stained using rhodamine phalloidin at 4 h post-scratch. As seen previously [9], fibroblasts cultured on native collagen increased their filopodia by 4 h. In contrast, fibroblasts grown on 3DG-collagen showed minimal extension of their filopodia at 4 h (Figure 3). Inhibition of p38 MAPK with SB202190 delayed filopodia extension into the wound site when fibroblasts were cultured on native collagen. However, inhibition of p38 MAPK induced filopodia extension in fibroblasts cultured on 3DG-collagen (Figure 3). These results suggest that when fibroblasts are cultured on native collagen p38 MAPK is required for proper filopodia extension, while upregulation of p38 MAPK in fibroblasts grown on 3DG-collagen leads to inhibition of filopodia extension. These results corroborate the current findings that p38 MAPK differentially regulates cellular migration, which is dependent upon the dermal fibroblast's interaction with extracellular stimuli. Moreover, these results are consistent with the findings that p38 MAPK reduces cell migration through downregulation of the phosphorylation of AKT when fibroblasts are cultured on 3DG-collagen as this kinase is needed for proper migration and cell survival.

### Fibroblast proliferation is dependent upon p38 MAPK activation of AKT

**Figure 4:**
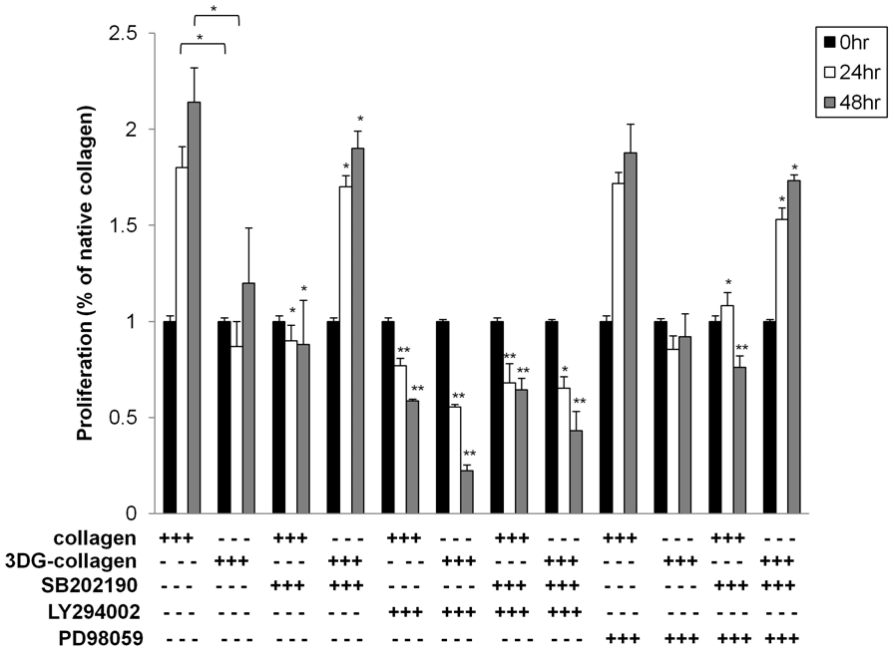
Proliferation rates in fibroblasts pretreated with kinase inhibitors. 4×10^3^ cells/well were pretreated with the p38 MAPK inhibitor SB202190, AKT inhibitor LY294002, or ERK1/2 inhibitor PD98059 for 1 h and then seeded in a 96-well plate containing either native collagen or 3DG-collagen for 0 h, 24 h, and 48 h. At each designated time point the level of proliferation was determined using the cell proliferation reagent WST-1. Quantification of each sample was determined by measuring the absorbance at 450 nm, with a reference wavelength of 600 nm. Comparisons were performed within each time point and compared to their respective controls (native collagen or 3DG-collagen). Data are mean ± SD (n = 3), **P<0.01, *P<0.05.

AGE-modified collagen can reduce the proliferation of cells [37], [38] Both ERK1/2 and AKT are known kinases integrally involved in cell proliferation [14], . Because p38 MAPK has been shown to inversely regulate the expression of these kinases in both a stress and growth environment, we investigated the role of p38 MAPK in cell proliferation. Fibroblasts were pretreated with SB202190, LY294002, PD98059, or a combination of the inhibitors and cultured on either native collagen or 3DG-collagen. Cell proliferation was measured at 0 h, 24 h, and 48 h. Fibroblasts cultured on native collagen were found to steadily proliferate over 48 h, while fibroblasts cultured on 3DG-collagen decreased their proliferative capacity by 48 h (Figure 4, p<0.05). There was a downregulation in the rate of proliferation when p38 MAPK was inhibited in fibroblasts cultured on native collagen compared to control. When p38 MAPK was inhibited with SB202190 in fibroblasts cultured on 3DG-collagen, proliferation was restored (Figure 4, p<0.05) to numbers similar to fibroblasts cultured on native collagen at 48 h.

Next we investigated which protein kinase p38 MAPK was regulating to induce proliferation. Fibroblasts were pretreated with the ERK1/2 inhibitor PD98059 or the AKT inhibitor LY294002 and proliferation was measured over 48 h. We found that the rate of proliferation in fibroblasts cultured on native collagen was dependent on AKT but not ERK1/2 as only inhibition of AKT showed similar levels of proliferation to that seen in fibroblasts pretreated with the p38 MAPK inhibitor (Figure 4, p<0.01). Moreover, the simultaneous inhibition of p38 MAPK and AKT did not provide any significant additive effect to the decreased rate of proliferation seen in these fibroblasts, suggesting that proliferation is dependent on the p38 MAPK regulation of AKT (Figure 4, p<0.01). In addition, fibroblasts pretreated with only the AKT inhibitor for 1 h and then cultured on 3DG-collagen showed no signs of proliferation in the first 24 h and by 48 h the majority of the cells had died, while there was no significant change in the proliferation capacity of these cells when ERK1/2 was inhibited (Figure 4, p<0.01). These studies suggest that 3DG-collagen-induced p38 MAPK downregulates cell proliferation by decreasing phosphorylation of AKT.

### AKT activation is required for regulation of caspase-3 by p38 MAPK in fibroblasts cultured on native collagen and 3DG-collagen

**Figure 5:**
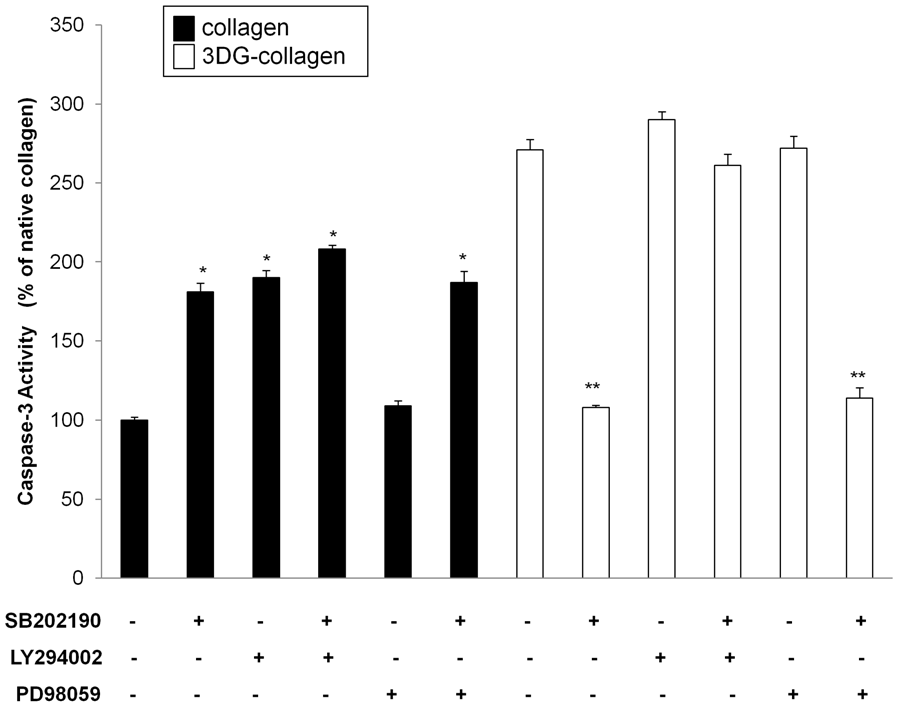
Caspase-3 activation after fibroblast pretreatment with kinase inhibitors. Fibroblasts were pretreated with the inhibitors; p38 MAPK inhibitor SB202190, AKT inhibitor LY294002, or ERK1/2 inhibitor PD98059 for 1 h and then cultured on native collagen or 3DG-collagen for 24 h. Whole cell lysates were assayed for caspase-3 activity at an absorbance of 405 nm. All comparisons were made against their respective controls (native collagen or 3DG-collagen alone). Data are mean ± SD (n = 3), **P<0.0005, *P<0.001.

ASK1 is able to phosphorylate p38 MAPK to signal the activation of apoptotic cascade [15], [27], [39], [40]. However, recent studies have shown that H-ras-dependent upregulation of p38 MAPK can enhance cell survival in cancer cells through activation of AKT [21], [22], [23], [24]. Furthermore, we demonstrated that 3DG-collagen induced the expression of caspase-3 [11]. Therefore, we investigated the role of p38 MAPK in caspase-3 activation under growth and stress conditions. When fibroblasts were pretreated with the p38 MAPK inhibitor SB202190 and cultured on native collagen, there was a 81%±5.5% increase in the level of caspase-3 activation (Figure 5, p<0.001). To determine how p38 MAPK is regulating caspase-3 activation in fibroblasts cultured on native collagen we investigated the role of AKT and ERK1/2. The regulation of caspase-3 by p38 MAPK in fibroblasts cultured on native collagen was dependent on the activation of AKT as inhibition of AKT increased the level of caspase-3 to 190%±4.5% (p<0.001). Additionally, there was no significant additive effect on the level of caspase-3 activation when both p38 MAPK and AKT were simultaneously inhibited (208%±2.6%). Moreover, the inhibition of ERK1/2 with PD98059 did not significantly increase the expression of caspase-3 (109%±3.2%), while inhibition of both ERK1/2 and p38 MAPK increased the expression of caspase-3 to 187%±7.0% (Figure 5, p<0.001). Survival of fibroblasts cultured on native collagen is dependent upon p38 MAPK-induced AKT activation.

We next investigated the role of p38 MAPK in fibroblasts cultured on 3DG-collagen. Fibroblasts cultured on 3DG-collagen increased the expression of caspase-3 to 271%±6.6% and this upregulation was abrogated when p38 MAPK was inhibited (Figure 5, 108%±1.5%, p<0.0005). Also, p38 MAPK downregulated the phosphorylation of AKT and ERK1/2 in fibroblasts cultured on 3DG-collagen (Figure 1); therefore, we investigated whether downregulation of AKT or ERK1/2 by p38 MAPK was responsible for increased caspase-3 activation in fibroblasts cultured on 3DG-collagen. The activation of caspase-3 by p38 MAPK in fibroblasts cultured on 3DG-collagen was shown to be dependent on the inactivation of AKT. Inhibition of AKT (LY294002) caused a 190%±5.0% increase in the level of caspase-3 activation in fibroblasts cultured on 3DG-collagen (Figure 5). This increase was not significantly altered when fibroblasts were pretreated with both AKT and p38 MAPK inhibitors simultaneously (161%±7.1% increase), unlike that seen in fibroblasts treated simultaneously with ERK1/2 (PD98059) and p38 MAPK (SB202190) inhibitors (172%±6.8% increase with ERK1/2 inhibitor vs. 14%±6.6% increase with ERK1/2 and p38 MAPK inhibitor). These results suggest that in fibroblasts cultured on 3DG-collagen p38 MAPK reduces the phosphorylation of AKT, which is responsible for increased caspase-3 activation.

### Type I collagen expression is inversely regulated by p38 MAPK in fibroblasts cultured on native collagen and 3DG-collagen

**Figure 6:**
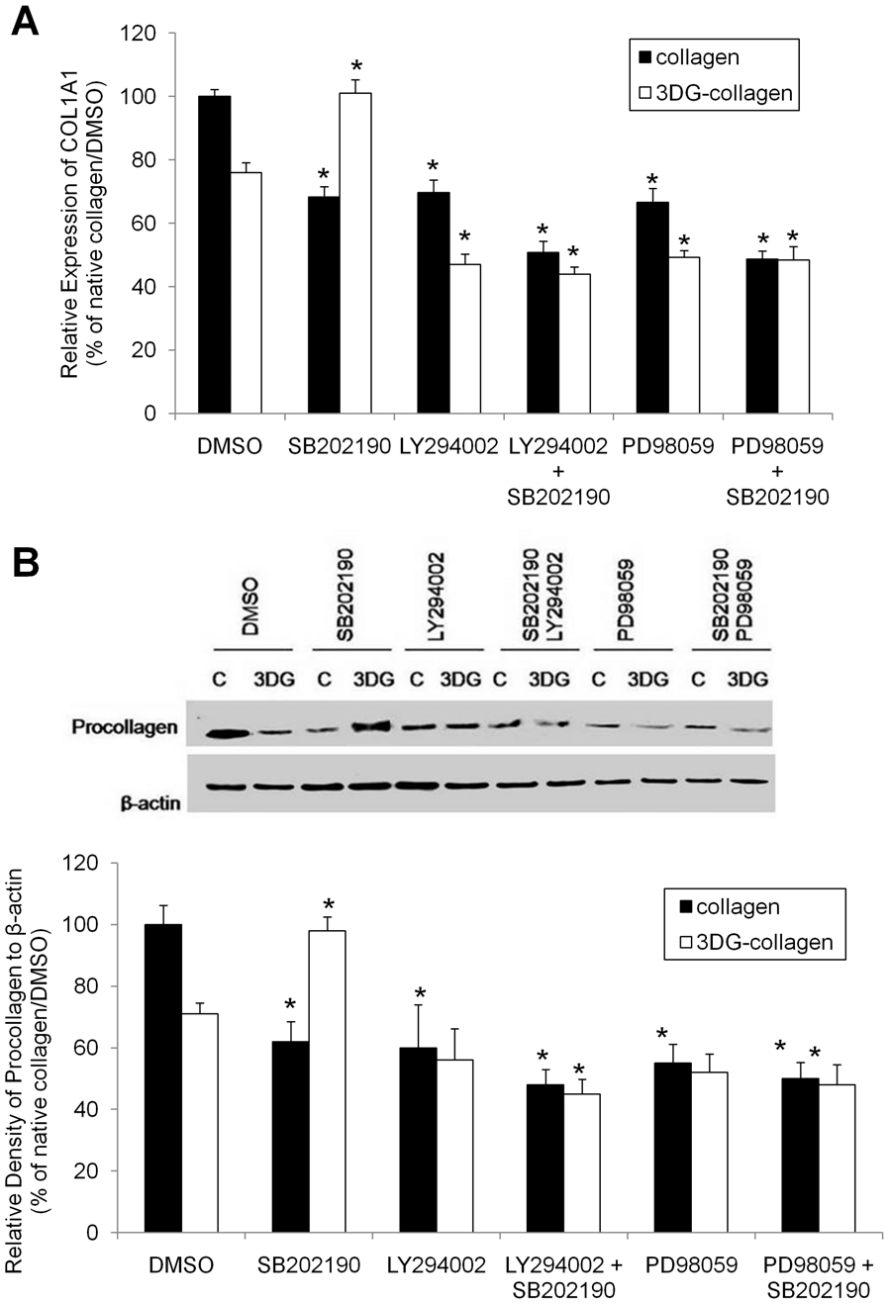
Expression of type I collagen after fibroblast pretreatment with kinase inhibitors. Fibroblasts were pretreated with p38 MAPK inhibitor (SB202190), AKT inhibitor (LY294002), or ERK1/2 inhibitor (PD98059) for 1 h and then cultured on native collagen or 3DG-collagen for 24 h. **A**, COL1A1 mRNA levels were quantified by real-time RT-PCR and transcripts were normalized to -actin. **B**, The bar graph was obtained by determining the expression levels of procollagen analyzed by Western blot and normalized to -actin which was the loading control and the bars correspond to the relative density of procollagen protein compared to that observed in cells cultured on native collagen treated with DMSO. Statistical comparisons were also made against their respective controls (native collagen or 3DG-collagen). Data are mean ± SD (n = 3), *P<0.05.

During wound healing, type I collagen is synthesized by dermal fibroblasts to aid in successful contraction of the wound margins [1], [2]. We previously demonstrated that 3DG-collagen inhibits the expression of type I collagen in fibroblasts [10]; therefore, we investigated the role of p38 MAPK in type I collagen production. Fibroblasts were pretreated for 1 h with the inhibitors SB202190, LY294002, PD98059, or a combination of inhibitors were cultured on native collagen or 3DG-collagen for 24 h. Fibroblasts cultured on native collagen induced the expression of collagen at both the level of transcription and translation. In contrast, fibroblasts cultured on 3DG-collagen reduced both the transcript levels of COL1A1 (75%±3.2%, Figure 6A) and the protein levels of procollagen (71%±3.6%, Figure 6B). Inhibition of p38 MAPK with SB202190 in fibroblasts cultured on native collagen showed both reduced transcript levels of COL1A1 (68%±3.2%, p<0.05) and reduced expression of procollagen (62%±6.5%, p<0.05, Figure 6). In contrast, inhibition of p38 MAPK restored the expression of COL1A1 (101%±4.2%) and procollagen (98%±4.4%) in fibroblasts grown on 3DG-collagen (Figure 6, p<0.05).

Moreover, p38 MAPK regulation of collagen was dependent on the activation of both AKT and ERK1/2 as there was a similar decrease in collagen expression when both p38 MAPK and AKT and p38 MAPK and ERK1/2 were inhibited in fibroblasts grown on native collagen (Figure 6, p<0.05). In fibroblasts cultured on 3DG-collagen, collagen expression was dependent on the p38 MAPK downregulation of both AKT and ERK1/2 as restoration of collagen expression by p38 MAPK inhibition was abrogated when both AKT and ERK1/2 were independently inhibited (Figure 6, p<0.05). These data suggest that p38 MAPK is playing a major role in the transcription and translation of collagen. Furthermore, this data highlights that p38 MAPK is playing a positive role in the regulation of collagen when fibroblasts are grown on native collagen, while playing a negative role in fibroblasts grown on 3DG-collagen.

## Discussion

Cutaneous wound healing is a dynamic process that is regulated by the balance of the pro-survival signaling pathway and the pro-apoptotic signaling pathway [1], [2]. During normal wound repair the dermal fibroblasts proliferate and migrate allowing for contraction of the wound margins [2], [4]. However, during times of chronic wound repair as seen in diabetes, the dermal fibroblast appears to be incapable of proper proliferation and migration resulting in aberrant and delayed wound closure [3], [4], [5], [6], [7], [8]. Moreover, there is an increase in the number of apoptotic cells residing in chronic wounds, which would further decrease the wound healing rate [6], [7], [8]. Recent evidence has provided a dual role for the stress-activated kinase p38 MAPK in both normal and chronic wound repair and our findings support this observation. Under normal wound repair, studies have shown that p38 MAPK can be activated to induce cell proliferation and migration; while p38 MAPK has been shown to induce apoptosis and prevent cell migration in chronic diabetic wounds [1], [14], [20], [22], [25], [41], [42]. In support of this, increased levels of 3DG-collagen have been shown to induce caspase-3 and reduce fibroblast migration [9], [11]; however, the signaling mechanism by which 3DG-collagen may impact diabetic wound healing had not been investigated. These results suggest that p38 MAPK is being differentially regulated by an upstream mediator to induce either cell growth or cell stress in normal wounds and chronic wounds, respectively. Because of the antagonizing role p38 MAPK plays, we sought to better understand how p38 MAPK signals to promote cell survival and cell death in normal and chronic wounds.

In this study, we provide evidence for a novel mechanism by which the differential regulation of p38 MAPK is dependent upon the fibroblast's interaction with varying extracellular stimuli. p38 MAPK can be regulated by both the growth factor induced-GTPase H-ras and the pro-apoptotic kinase ASK1. Previous studies have provided a fundamental role for H-ras-induced p38 MAPK signaling in promoting cell survival; while activation of ASK1 induces p38 MAPK signaling to promote apoptosis [15], [22], [23], [24], [27], [30], [43], [44]. Further evidence has suggested that the activation of H-ras can suppress the phosphorylation of ASK1 thereby preventing cell death and promoting proliferation and migration of cells [45], [46]. Through the use of pharmacological inhibitors, we found that fibroblasts cultured on 3DG-collagen have a sustained activation of p38 MAPK as evidenced by its upregulation at 24 h (Figure 1). Studies have shown that sustained activation of p38 MAPK is responsible for the increased apoptosis and reduced cellular migration [16]. This effect is dependent on the ability of p38 MAPK to be activated as a stress kinase by an upstream mediator such as ASK1. Also, ASK1 has been shown to induce p38 MAPK during times of cellular stress [39]. In contrast, native collagen transiently phosphorylates p38 MAPK to promote growth of the fibroblast, which suggests that under normal growth conditions p38 MAPK may be phosphorylated by the upstream mediator H-ras.

p38 MAPK cross-talks with other members of the MAPK family, especially ERK1/2 and AKT [29], [30]. Under normal conditions, signaling within the fibroblast via p38 MAPK, ERK1/2, and AKT results in proliferation and migration of the cell [14], [21]. However, under times of cellular stress, p38 MAPK may inhibit the activation of ERK1/2 and AKT leading to the upregulation of pro-apoptotic genes [16], [31], [32]. 3DG-collagen induced changes in the signaling cross-talk between p38 MAPK and ERK1/2 and AKT, as we observed that 3DG-collagen decreased the phosphorylation of ERK1/2 and AKT, and this was found to be dependent on p38 MAPK activation (Figure 1). Additionally, inhibition of p38 MAPK in fibroblasts cultured on native collagen reduced the phosphorylation of ERK1/2 and AKT, which supports previous claims [14], [17] that under normal conditions p38 MAPK, ERK1/2, and AKT work synergistically to enhance cell growth (Figure 1).

Downregulation of the ERK1/2 and AKT pathway can lead to decreased migration and proliferation of the cell [14], [16], [17], [21], [31], [32], [47]. Previous work in our laboratory has demonstrated that 3DG-collagen decreased the proliferation and migration of fibroblasts [9] suggesting that decreased phosphorylation of ERK1/2 and AKT mediated via p38 MAPK activation is responsible for this growth depression. Chemical inhibition of p38 MAPK with SB202190 in fibroblasts grown on 3DG-collagen restored the migration potential of these fibroblasts to that observed in fibroblasts grown on native collagen (Figure 2). The decreased migration seen in fibroblasts cultured on 3DG-collagen was due to the p38 MAPK depression of phosphorylated AKT (Figure 2). Also, p38 MAPK inhibition in fibroblasts grown on native collagen reduced the migratory capacity of these cells, supporting a dual role for the need of both p38 MAPK and AKT activation in non-stressed cells (Figure 2). In support of this, the AKT inhibitor did not affect the phosphorylation state of p38 MAPK suggesting that the p38 MAPK inhibitor can act on both the activity of itself and that of AKT, while the AKT inhibitor can only act by reducing AKT phosphorylation. Although inhibition of ERK1/2 did not significantly alter the migration rate of fibroblasts, we cannot rule out the possibility that ERK1/2 plays a role in migration as AKT activation could mask the effects of ERK1/2. Furthermore, simultaneous inhibition of both p38 MAPK and AKT in fibroblasts cultured on 3DG-collagen did not result in reduced migration comparable to that seen when AKT alone was blocked, suggesting that ERK1/2 activation is playing a role in controlling fibroblast migration. This role however, may be secondary to that of AKT.

During wound healing, fibroblasts along the wound edge begin to extend their filopodia to promote migration into the wound. We previously demonstrated that at 4 h post-wounding, fibroblasts cultured on 3DG-collagen show minimal extension of their filopodia into the wound [9], in contrast to the extension of filopodia observed in fibroblasts cultured on native collagen. Filopodia extension was restored in fibroblasts cultured on 3DG-collagen when p38 MAPK was inhibited (Figure 3). Together these results support the idea that p38 MAPK promotes cell migration by upregulating AKT in fibroblasts cultured on native collagen, while p38 MAPK acts as a stress kinase to depress migration in fibroblasts cultured on 3DG-collagen.

Proliferation of fibroblasts also showed dependence of p38 MAPK induction of AKT. p38 MAPK-induced AKT activity promoted cell proliferation in fibroblasts cultured on native collagen (Figure 4). Moreover, 3DG-collagen reduced the proliferation of fibroblasts, which was found to be dependent on p38 MAPK suppression of AKT (Figure 4), which supports a dual role for p38 MAPK controlling wound closure. Accompanying reduced proliferation in fibroblasts cultured on 3DG-collagen was an upregulation in caspase-3 activation. Caspase-3 is an early marker of apoptosis, which can be activated by ASK1-induced p38 MAPK. Upregulation of caspase-3 in fibroblasts cultured on 3DG-collagen was dependent on the p38 MAPK downregulation of AKT (Figure 5). In contrast, p38 MAPK promoted cell survival through phosphorylation of AKT in fibroblasts cultured on native collagen (Figure 5). These results further suggest native collagen promotes cell survival through activation of p38 MAPK, while 3DG-collagen results in caspase-3 activation through activation of p38 MAPK. The final step in wound closure is the fibroblast's ability to remodel the ECM through production of type I collagen. p38 MAPK promoted the production of collagen both transcriptionally and translationally in fibroblasts cultured on native collagen, which was found to be dependent on the activation of AKT and ERK1/2 by p38 MAPK (Figure 6). In contrast, activation of p38 MAPK reduced the production of collagen, which was dependent on the downregulation of AKT and ERK1/2, in fibroblasts cultured on 3DG-collagen (Figure 6). Because there was an additive effect when both p38 MAPK and ERK or AKT were inhibited, p38 MAPK, ERK, and AKT could be working through independent but redundant pathways. Although this claim is beyond the scope of this paper, it may be worth investigating other upstream mediators that control p38 MAPK, ERK, and AKT simultaneously such as TGF-beta, H-ras, and ASK-1, which are known to be involved in wound healing. Wound healing is a complex process that utilizes a network of signaling pathways activated by different stimuli and can converge upon multiple kinases to regulate gene expression, proliferation, and survival of the cell. Because both H-ras and ASK1 have been shown to activate p38 MAPK during times of growth and stress, respectively, we anticipate that H-ras mediates the phosphorylation of p38 MAPK to promote migration and proliferation of the fibroblast, while ASK1 phosphorylates p38 MAPK to activate downstream stress responses.

**Figure 7:**
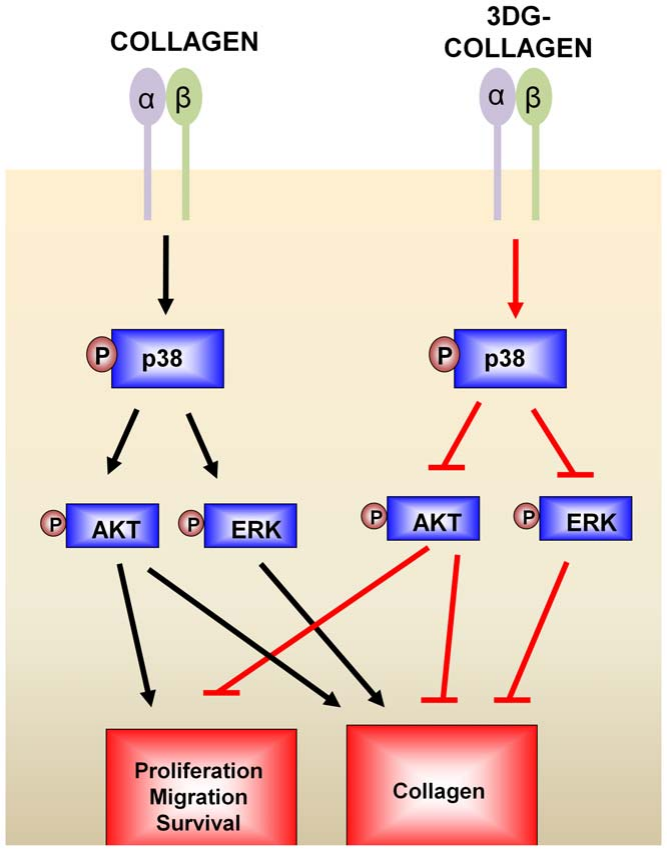
Diagram of p38 MAPK regulation of wound healing on native collagen and 3DG-collagen. Under normal wound healing phosphorylation of p38 MAPK activates AKT and ERK1/2 to promote migration, proliferation, and collagen production. During diabetic wound healing, 3DG-collagen negatively impacts the dermal fibroblast by phosphorylating p38 MAPK to downregulate the expression of ERK1/2 and AKT and promote reduced cell proliferation and migration.

The antagonizing roles of p38 MAPK in wound healing have not been extensively elucidated. Furthermore, the signaling cross-talk among p38 MAPK and other kinases such as AKT and ERK1/2 have not been fully investigated in dermal fibroblasts cultured on both native collagen and 3DG-collagen. Here we report that under normal wound healing conditions p38 MAPK promotes the closure of the wound through increased fibroblast migration, proliferation, and collagen production. Activation of p38 MAPK as a growth kinase resulted in upregulation of AKT and ERK1/2 which were essential for proliferation and migration of fibroblasts on native collagen (Figure 7). In contrast, fibroblasts cultured on 3DG-collagen, decreased wound closure similar to that seen in chronic diabetic wounds (Figure 7). p38 MAPK has been implicated as a stress kinase in chronic wounds, which results in decreased proliferation and migration and increased apoptosis of fibroblasts [42]. Although these studies relied heavily upon the use of pharmacological inhibitors, we did not notice any change in the phosphorylation of p38 MAPK following the use of AKT and ERK inhibitors (Figure 2B). However, it should be noted that other non-specific off target effects may have taken place and future studies should also include siRNA against p38 MAPK to validate these claims. Despite the reliance on pharmacological inhibitors, the data presented does corroborate the different roles that p38 MAPK has been shown to play during times of stress and growth.

Regulation of p38 MAPK activation appears to be critical during wound healing. This apparent dichotomy between normal and chronic wound healing associated with p38 MAPK suggests that extracellular stress on the fibroblast can alter the signaling cross-talk associated with cell survival to promote pro-apoptotic events. More importantly, this is the first evidence providing a mechanism by which 3DG-modified collagen, which is upregulated in diabetic wounds, prevents *in vitro* wound closure through the dysregulation of p38 MAPK. These results may have bearing on current therapeutic strategies for treating wounds in diabetic patients.

## Materials and Methods

This study was approved by the Internal Review Board of Drexel University for human studies.

### Tissue Culture

Collagen coating of culture dishes with cross linking of the collagen has been described previously [9], [11]. Normal human dermal fibroblasts from individuals (GM08333, GM00498, GM04190, GM00321) aged 3-85years old (passage <10) were purchased from the Coriel Institute (Camden, NJ). Fibroblasts were seeded onto native collagen and 3DG-collagen coated dishes and cultured until 70% confluent in Dulbecco's Modified Eagle's Medium (DMEM) supplemented with 10% dialyzed FBS and 1% penicillin/streptomycin unless otherwise noted.

### Chemicals and Antibodies

p38 MAPK inhibitor SB202190 was purchased from Sigma (St. Louis, MO). AKT inhibitor LY294002, ERK1/2 inhibitor PD98059, and monoclonal antibody against type I collagen (sc-133179) was purchased from Santa Cruz Biotechnology (Santa Cruz, CA). Polyclonal antibodies against phospho-p38 MAPK, phospho-ERK1/2, phospho-AKT, total p38 MAPK, total ERK1/2, total AKT, and *β*-actin were purchased from Cell Signaling Technologies (Danvers, MA). Secondary antibodies were purchased from Jackson Labs (West Grove, PA).

### Inhibition of p38 MAPK, ERK1/2, and AKT

For inhibition studies, fibroblasts were cultured until 70% confluent, trypsinized, preincubated for 1 h with or without the p38 MAPK inhibitor SB202190 (10 µM), ERK1/2 inhibitor PD98059 (50 µM), or AKT inhibitor LY294002 (25 µM), and then replated onto collagen or 3DG-collagen coated dishes for 24 h in DMEM containing 1% FBS and 1% Pen/Strep. The concentrations of inhibitors are similar to doses used in previously published studies [19], [23], [48].

### SYBR Green Quantitative RT-PCR

Cells were harvested and RNA was extracted using the RNeasy Mini kit (Qiagen, Valencia, CA) according to manufacturer's protocol. To verify expression of COL1A1; 2.0 µg of total RNA was reverse-transcribed using Superscript-III reverse transcriptase (Invitrogen Carlsbad, CA), according to manufacturer's protocol. Transcripts were quantified using SYBR green PCR amplification (Qiagen). All mRNA transcripts were normalized to *β*-actin expression. The following primers were employed to detect transcripts of interest: COL1A1-forward: 5′-CCAGAAGAACTGGTACATCAGCA-3′ and COL1A1-reverse: 5′-CGCCATACTCGAACTGGAAT-3′; *β*-actin-forward 5′-TTGCCGACAGGATGCAGAA-3′ and *β*-actin-reverse 5′-GCCGATCCACACGGAGTACTT-3′.

### Western blotting

Cells were harvested and protein was extracted using cell lysis buffer supplemented with 0.3% PMSF and proteinase and phosphatase inhibitors. 100 µg of protein from each sample was size fractionated on 10% SDS PAGE gels or 4–12% NATIVE PAGE gel (Invitrogen) and transferred to PVDF membrane. The membrane was blocked with 5% skim milk or 3% BSA with antibodies directed at phospho-groups, and probed with an antibody directed against either procollagen (1∶200), *β*-actin (1∶1000), phospho-ERK1/2 (1∶1000), phospho-p38 MAPK (1∶1000), phospho-AKT (1∶1000), total p38 MAPK (1∶1000), total AKT, or total ERK1/2 (1∶1000). The membrane was washed with TBS-Tween and incubated with a secondary antibody, goat-anti-rabbit-HRP (1∶2000) (Jackson Labs, West Grove, PA). The signal was developed with SuperSignal Chemiluminescent Substrate (Pierce, Rockford, IL). The bands were assessed by densitometry by Image J software (NIH).

### *In vitro* Scratch Assay

Wound healing assays were performed as previously described [9]. Confluent fibroblasts cultured on native collagen or 3DG-collagen were pretreated with 10 µM of SB202190, 25 µM of LY294002, 50 µM of PD98059, or the vehicle DMSO for 1 h. Scratch wounds were introduced with a sterile pipette tip onto the confluent monolayer of fibroblasts. The cells were washed to remove damaged and detached cells and DMEM containing 1% FBS was added to prevent proliferation. At the time of wounding, inhibitors were added back into the media containing 1% FBS. The wound area was photographed immediately after wound induction (0 h), and again at 24 h and 48 h post scratch using brightfield exposure at 10 X magnification on a Nikon eclipse 80i epi-fluorescence microscope. The images were captured using an RT3 Color Mosaic Camera (Diagnostic Instruments, Sterling Heights, MI). The distance between the edges of the wound were measured at ten different areas from the wound edge to edge using Spot software. The measurements were then converted into a percentage using the formula: % of wound remaining  =  (measurement at time X/measurement at time 0 h) * 100; then to obtain the % of wound closure: 100% - % of wound remaining.

### Immunofluorescence of Filopodia

For F-actin staining of filopodia extension, fibroblasts were wounded as described above, washed three times with sterile PBS, and air-dried at 4 h post scratch. The fibroblasts were then fixed with 4% paraformaldehyde, permeabilized with 0.3% Triton X-100, and stained with rhodamine phalloidin (Cytoskeleton, Denver, CO). The samples were counterstained with DAPI (Vector Laboratories, Burlingame, CA). Five images from each sample were captured as described above at 20 X and 40 X magnification.

### Proliferation Assay

Cells were plated at a density of 4×10^3^ cells/well on a native collagen or 3DG-collagen coated 96-well plate in 100 µL of complete DMEM supplemented with 10% FBS and 1% Penn/Strep. Proliferation of cells was measured at 0 h, 24 h, and 48 h. Proliferation was measured by the addition of Cell Proliferation Reagent WST-1 to cells for 4 h at 37°C. Absorbance of the samples was measured at 450 nm with a reference wavelength of 600 nm according to the manufacturer (Roche, Indianapolis, IN).

### Caspase-3 Assay

Cells were harvested and lysed in cell lysis buffer as previously described above. Whole cell lysates were combined with Caspase-3 substrate reaction buffer and incubated for 3 h at 37°C and the absorbance was measured at 405 nm with a plate reader according to the manufacturer (Assay Designs, Ann Arbor, MI). Background readings from cell buffers and substrates were subtracted from the sample readings according to the protocol.

### Statistical Analysis

The data are presented as mean ± SD. The resulting data were subjected to either a two-tailed unpaired Student *t*- test for comparison between two groups, or a one-way ANOVA for comparison between multiple groups followed by Tukey's post-hoc test with GraphPad InStat 3 software (San Diego, CA). P values <0.05 were considered significant.
